# Th22 cells promote the transition from homeostatic to reactive microglia in diabetic encephalopathy

**DOI:** 10.1007/s00592-024-02384-0

**Published:** 2024-12-04

**Authors:** Sheng-Xue Yu, Hong Dan Yu, Yu-Fei Wang, Tie-Feng Yao, Song-Ze Lv, Yan-Chuan Wang, Jun-Qi Li, Wen-Qiang Liu, Jia-Yuan Ding, Xue-Zheng Liu, Zhong-Fu Zuo, Wan-Peng Liu

**Affiliations:** 1 Liaoning Key Laboratory of Diabetic Cognitive and Perceptive Dysfunction, Jinzhou Medical University, Jinzhou, China; 2 Department of Anatomy, Histology and Embryology, Jinzhou Medical University, Jinzhou, China; 3 Department of First Clinical College, Jinzhou Medical University, Jinzhou, China

**Keywords:** Diabetic encephalopathy, Microglia, Th22, IL-22

## Abstract

**Background:**

Diabetic encephalopathy (DE) is one of the most serious complications of diabetes mellitus (DM), and its pathogenesis has not yet been clarified. Th22 cells are a newly discovered class of CD4^+^ T cells that play important roles in inflammatory, autoimmune and infectious diseases. However, it is unclear whether Th22 cells are involved in the pathogenesis of DE.

**Methods:**

We established a T2DM mouse model in vivo and cocultured Th22 cells with microglia under high glucose (HG) conditions in vitro. Cognitive dysfunction was evaluated using the *Morris water maze (MWM*) test; blood‒brain barrier (BBB) integrity was evaluated using the Evans blue (EB) extravasation assay; Th22 cells and IL-22 receptors were detected by immunofluorescence; and IL-1β, TNF-α, iNOS, CD86, Arg-1, and CD206 protein expression was measured by *Western Blot (WB)* analysis.

**Results:**

Th22 cells passed through the BBB into the hippocampus and secreted interleukin-22 (IL-22), and the mice subsequently exhibited decreased learning and memory abilities. In the DE model, IL-22 promoted the transformation of homeostatic microglia into reactive microglia as well as the inflammatory response. Additionally, coculture of Th22 cells with BV2 microglia cultured under HG conditions increased the production of proinflammatory cytokines, and the microglia showed reactive changes. Mechanistically, IL-22Rα1 acted as a ligand, and IL-22 bound to IL-22Rα1 on microglia to drive primary microglia-induced inflammatory responses. Interestingly, interleukin-22 binding protein (IL-22BP) directly binds to IL-22Rα1 on microglia to inhibit the proinflammatory effects of IL-22.

**Conclusion:**

Th22 cells secrete IL-22 after passing through the BBB into the hippocampus and promote the transformation of homeostatic microglia into reactive microglia, which induces an inflammatory response, exacerbates learning and memory impairment and cognitive deficits, and contributes to and accelerates the development of DE.

## Introduction

Diabetes mellitus (DM) is an immune and metabolic disease characterized by disordered glucose and lipid metabolism caused by a variety of genetic, metabolic, and immune factors [1, 2]. Diabetic encephalopathy (DE) is a serious complication of DM and is a form of diabetes-induced neuropathy characterized by neuroinflammation and aberrant synaptogenesis in the hippocampus, leading to cognitive decline and, in severe cases, to dementia [3–5]. Recent evidence suggests that the pathogenesis of DM is related to immune mechanisms, including abnormalities in cellular and humoral immunity [6–8]. CD4^+^ helper T cells (T helper cells, Th) play an important role in the immune response [9, 10]. CD4^+^ T cells are thought to have a “supportive” role in the immune system since, to a large extent, CD4^+^ T cells act mainly through indirect cellular contacts in vivo via cytokines [11, 12]. In diabetes, abnormally activated CD4^+^ T cells increase the expression of peripheral blood inflammatory factors by secreting the cytokines interleukin-17 (IL-17) and interferon-γ (IFN-γ) [13]. Recent research suggests that not only Th1, Th2, Th17, Th9, and Th17 subsets but also the Th22 subset of CD4^+^ T cells are involved in inflammatory and autoimmune disorders [14, 15]. Although a previous study showed that Th22 cells are associated with DE, the exact underlying mechanism is still unknown.

Th22 cells primarily produce interleukin-22 (IL-22), a potent cytokine that regulates many key cellular pathways in tissues. Interleukin-22 binding protein (IL-22BP) is a soluble secreted single-chain receptor that lacks both trans- and intracellular structural domains. IL-22BP has a strong affinity for IL-22 [16]. Studies have shown that the affinity of IL-22 for IL-22BP exceeds that for its cell membrane-associated receptor, IL-22Rα1, by a factor of > 20 or > 1000 [17]. The strong affinity of IL-22 for IL-22BP and the binding mode between IL-22 and IL-22BP confirm that the binding of IL-22BP to IL-22 effectively inhibits the effects of IL-22 on cells.

Microglia are resident immune cells in the central nervous system. Microglia are highly dynamic and plastic cells that display various morphological/ultrastructural, transcriptional, metabolic, and functional states in the CNS under both healthy and pathological conditions [18]. Microglia are referred to as homeostatic microglia under basal conditions and as “reactive” or “responsive” microglia when describing their response to specific signals or under experimental conditions. However, *CNS* injury or stress leads to the initiation and proliferation of microglia, an increase in reactive microglia, maturation, and subsequently a series of inflammatory reactions induced by reactive microglia [19–21]. Reactive microglia are closely associated with neuroinflammatory diseases such as diabetes, Alzheimer’s disease, and cerebral ischemia [22, 23]. Blood brain barrier (BBB) is a protective mechanism that maintains homeostasis in the brain and protects the central nervous system against foreign bodies [24]. In vitro and in vivo studies have shown that DM can lead to impaired BBB integrity and subsequently increased BBB permeability [25, 26]. Microglia are among the components of the neurovascular unit (NVU) and are important for maintaining the structure and function of the BBB, but reactive microglia can damage the BBB [27, 28]. Under physiological conditions, microglia are in a homeostatic state and play an immunosurveillance role [29]. Rapid activation of microglia under pathological conditions is accompanied by adaptive changes in gene transcription [30]. Clinical studies have shown that reactive microglia can cause neuronal dysfunction, injury, and degeneration and play an important role in cerebrovascular diseases, neurodegenerative diseases, neurodevelopmental disorders, and psychiatric disorders [23, 31, 32]. Thus, modulating microglial status is a potential novel strategy for the treatment of DE.

Using a type 2 diabetes model, the present study focused on the immune mechanism of DE and explored the effect of Th22 cells on the state of microglia in DE through in vivo and in vitro experiments, as well as the specific mechanisms involved in the pathogenesis of DE, to provide new intervention targets for alleviating DE.

## Materials and methods

Six-week-old male C57BL/6 mice (weight 20 ± 3 g) were purchased from Beijing Huafukang Biotechnology Co., Ltd. (Beijing, China) and housed on a 12-h light and dark cycle (lights on at 7:00 a.m.) at a controlled room temperature for one week with ad libitum access to food and water. All the experimental protocols were approved by the Institutional Animal Care and Use Committee of Jinzhou Medical University. Written informed consent was obtained from the owners for the participation of their animals in this study.

### Experimental procedures

**Fig. 1:**
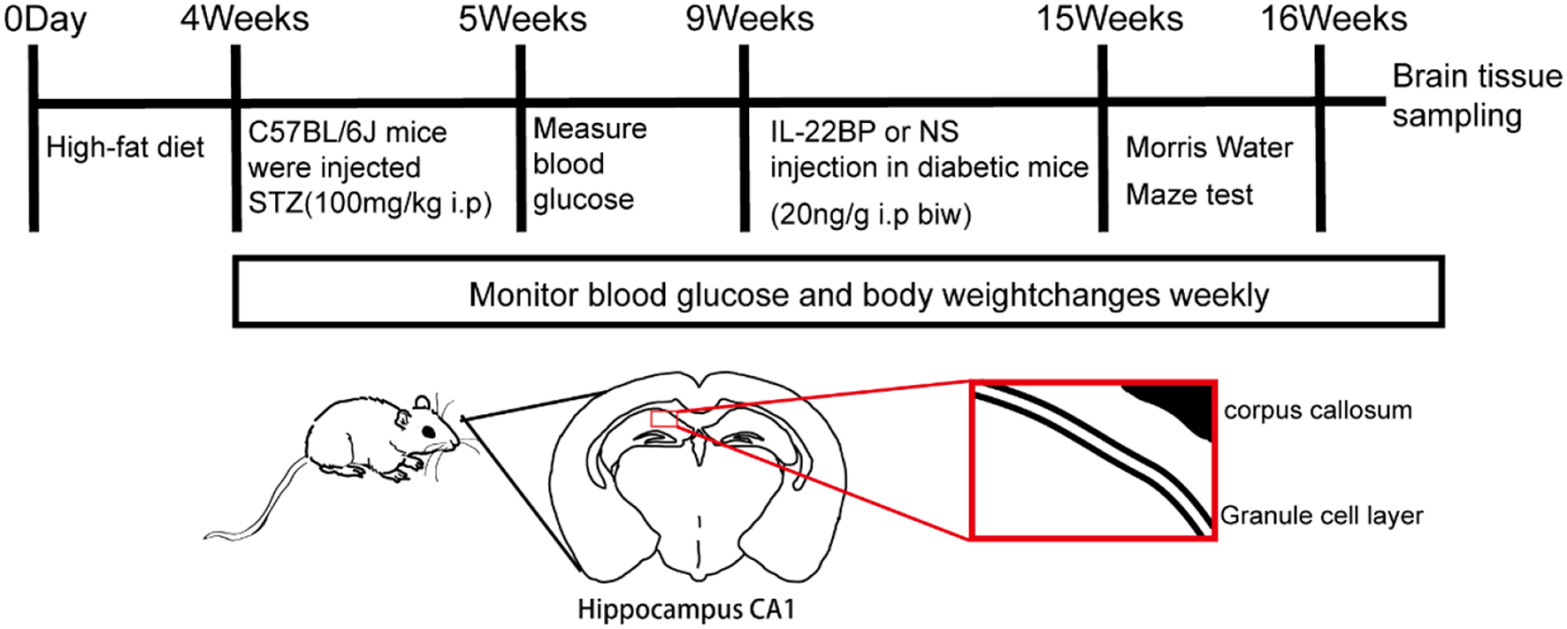
Schematic of the in vivo study performed following intraperitoneal injection of STZ

All mice were first fed an adaptive diet, and after 3 days, all mice except those in the control group were fed a high-fat and high-cholesterol diet (sunflower seeds and egg yolks added to standard chow). After 4 weeks of feeding, the mice were randomly divided into 4 groups: the control group (Con group, which included mice that received an intraperitoneal injection of citric acid buffer and an intraperitoneal injection of 0.9% saline) (*n* = 10); the diabetes group (DM group, which included mice that received an intraperitoneal injection of streptozotocin (STZ) and an intraperitoneal injection of 0.9% saline) (*n* = 10); the diabetes + recombinant IL-22BP protein group (IL-22BP group, which included mice that received an intraperitoneal injection of STZ and an intraperitoneal injection of recombinant IL-22BP protein (IL-22BP protein was dissolved in 0.9% saline) (*n* = 10); and the sham group (which included mice that received an intraperitoneal injection of citrate buffer and an intraperitoneal injection of 0.9% saline) (*n* = 10). After fasting and water deprivation for 12 h, all mice except those in the control group were intraperitoneally injected with 1.5% STZ (V900890; Sigma–Aldrich, dissolved in a freshly prepared 0.1 mol/L sodium citrate solution, pH = 4.5, 15 mg/mL, stored at low temperature in the dark) at a dose of 100 mg/kg. Blood glucose levels in whole blood collected from the tail vein were measured after 3 days. A blood glucose level ≥ 16.7 mmol/L was considered to indicate successful modeling of diabetes. Mice in the diabetes + IL-22BP group were injected intraperitoneally with IL-22BP (recombinant IL-22BP, 20 ng/g; 1 µg/ml; 9597-BP-050; PeproTech) twice/week for a period of 6 weeks beginning 1 month after modeling. 16 weeks later, the mice were sacrificed for brain tissue sample collection (Fig. 1).

### Morris water maze (MWM) test

The Morris water maze (MWM) test was used to test spatial learning and memory in the mice. The Morris water maze had a diameter of 120 cm and a height of 40 cm, and the platform had a diameter of 8 cm and a height of 15 cm. The water maze was located in the center of the room, and the walls of the room were blank except for a symmetrical light source. There were clear visual cues, and the pool was divided into four equal quadrants, i.e., the east, west, south, and north quadrants. The platform was fixed 1–2 cm below the water level in the target quadrant, and the water temperature was maintained at (20.0 ± 2) °C. Three days before the formal experiment, all the mice were acclimatized to the pool in the absence of the platform and allowed to swim freely for 60 s. The mice were removed and wiped dry immediately after training to avoid stress. The mice were placed in the pool from a randomly selected quadrant other than the target quadrant at a fixed time each day, and the escape latency (the time from when the mouse entered the water to when it found the hidden platform) was recorded. The mice were allowed to stay on the target platform for 10 s after they found it. If a mouse did not find the target platform within 60 s, the escape latency was recorded as 60 s, and the mice were guided to the target platform and kept there for 10 s. The mice were trained twice a day for 5 days. The swimming trajectory, escape latency, and time spent in the target quadrant were recorded using EthoVision XT 15 tracking software (EthoVision XT 9.0.726, Noldus). On day 6, the hidden platform was removed, and the spatial exploration experiment was conducted. The quadrant that previously held the platform was considered the target quadrant, and the mice were placed into the pool from this quadrant. The trajectories of the mice, the number of times they crossed the original platform location, and the time spent in the target quadrant over 60 s were recorded.

### Evans blue (EB) assay

BBB permeability was assessed by measuring Evans blue extravasation. EB dye (4% in 0.9% saline) was injected into the caudal vein (4 mL/kg). After the dye was allowed to circulate for 2 h, the mice were anesthetized with 1% pentobarbital sodium. After anesthesia, the chest cavity was opened, and 200–300 ml of normal saline (0.9% sodium chloride) was injected into the heart. After coronary perfusion, the head was severed, the brain was collected, and the hippocampus was dissected out. Half of the brain tissue was weighed, cut, incubated in dimethylformamide for 24 h, and centrifuged to obtain the supernatant. The absorbance at 620 nm was measured with a spectrophotometer. The other half of the brain tissue was frozen and sectioned into 10 μm sagittal sections with a freezing microtome, and EB extravasation was observed under a microscope.

### Immunofluorescence staining

After paraformaldehyde perfusion, the brain tissues were removed, dehydrated, and embedded in paraffin. Then, 4 μm paraffin sections were prepared and routinely dewaxed, hydrated, and subjected to antigen retrieval via high-pressure thermal repair. The brain tissues were washed in PBS, incubated in 0.3% Triton-100 in PBS for 30 min, washed in PBS, and incubated in 10% goat serum for 60 min at room temperature. Then, the tissues were incubated overnight at 4 °C with the following primary antibodies: anti-IL-22 (1:200; 5 µg/ml; Affinity), anti-IL-22Rα1 (1:200; 3.5 µg/ml; Proteintech), anti-AFX1 (1:100; 2 µg/ml; Santa Cruz), and anti-Iba1 (1:200; 3.5 µg/ml; Abcam). The sections were washed three times with PBS containing 0.1% Tween 20. The tissues were incubated with the corresponding secondary antibodies for 2 h at room temperature and stained with 4′,6-diamidino-2-phenylindole (DAPI; 0.5 µg/ml; Abcam, USA). The stained tissues were visualized via fluorescence microscopy (Olympus, Tokyo, Japan).

### Enzyme-linked immunosorbent assay (ELISA)

To study the effect of high glucose on IL-22 secretion by Th22 cells, the IL-22 levels in hippocampal and Th22 coculture cell supernatants were measured using ELISA kits according to the manufacturer’s instructions. The absorbance at 450 nm was measured via a zymograph. The concentration of IL-22 in the samples was calculated from a standard curve.

### Culture of BV2 microglia and Th22 cells

**Fig. 2:**
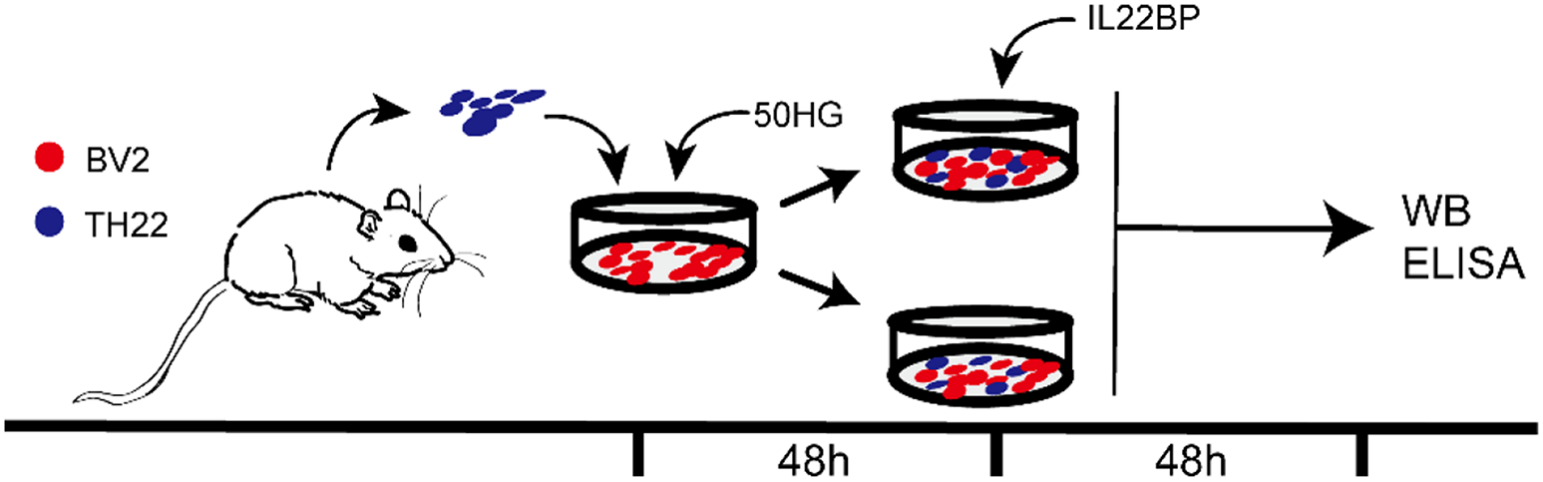
Coculture of Th22 cells with BV2 cells

BV2 cells (Procell) were cultured in Dulbecco’s modified Eagle medium (DMEM; Gibco, Life Technologies, Rockville, MD, USA) supplemented with 10% fetal calf serum, 100 µg/mL penicillin, and 100 µg/mL streptomycin in a humidified atmosphere of 5% CO_2_ at 37 °C. The complete medium was changed every 3 days, after which the cells were allowed to grow to 80% confluence. The spleens of C57BL/6J mice were removed and placed in Petri dishes containing Hanks’ solution to strip away the surrounding connective tissue, and the spleens were homogenized with a 5 ml syringe to prepare a single-cell suspension of mouse spleen cells. After filtration through a 70 μm filter, the spleen cells were centrifuged at 447.2 × g for 5 min at 4 °C. Naïve CD4^+^ T cells were isolated by negative selection using naïve CD4-bound microbeads (BioLegend). The cells were cultured in RPMI 1640 medium (Wako Pure Chemicals, Osaka, Japan) supplemented with 10% fetal bovine serum. Naïve CD4^+^ T cells (1 × 10^5^) were transfected with an anti-CD3 antibody (2 µg/mL), anti-CD28 antibody (0.5 µg/mL), or anti-IL-2 antibody (50 ng/mL). After 24 h, the Th22 cells were stimulated by adding recombinant mouse IL-6 (10 ng/mL) and recombinant mouse TNF-α (10 ng/mL) to the cell suspension. The expression of the markers IL-22 and AFX1 was analyzed by flow cytometry, and the results confirmed that the cells were Th22 cells. BV2 cells were cultured for 48 h as previously described. After 48 h, Th22 cells were added to BV2 cells in the presence of 50 mmol/L glucose, after which the plates were cultured for 48 h at 37 °C in a 5% CO2 atmosphere. The cells were then divided into 6 groups: the control group (complete DMEM), mannitol group (complete DMEM containing 50 mmol/L mannitol), HG group [33–35] (complete DMEM containing 50 mmol/L glucose), Th22 group (complete DMEM containing 50 mmol/L glucose and Th22 cells), IL-22BP group (complete DMEM containing 50 mmol/L glucose, Th22 cells, and IL-22BP), and sham group (complete DMEM containing 50 mmol/L glucose, Th22 cells, and saline) (Fig. 2).

### Immunoblotting

The total protein concentration was determined by a bicinchoninic acid (BCA) protein assay, and the samples were prepared and stored. A total of 30–50 µg of total protein was separated on a 10% SDS‒PAGE gel and subsequently transferred to a PVDF membrane. The sections were blocked in 5% BSA at room temperature for 2 h and incubated individually with primary antibodies against iNOS (1:1000; 0.6 µg/ml; rabbit, Proteintech), CD86 (1:1000; 0.5 µg/ml; rabbit, Proteintech), Arg-1 (1:5000; 0.16 µg/ml; rabbit, Proteintech), CD206 (1:5000; 0.2 µg/ml; rabbit, Proteintech), IL-1β (1:2000; 0.425 µg/ml; Proteintech), TNF-α (1:2000; 0.5 µg/ml; rabbit, Abcam), and β-actin (1:20000; 0.05 µg/ml; Proteintech) at 4 °C overnight (the primary antibodies were diluted in 0.1% BSA antibody diluent). The next day, the membrane was removed, washed well with TBS-Tween, and incubated with an HRP-labeled secondary antibody (1:5000) for 2 h at room temperature. The protein bands were visualized with a ChemiDoc Imaging System (Bio-Rad, USA). Finally, ImageJ software was used to measure the gray values of the protein bands and evaluate protein expression.

### Statistical analysis

All the statistical analyses were performed using GraphPad Prism. All the data are expressed as the mean ± SEM. Two-sample t tests were used for comparisons between two groups, and one-way or two-way analysis of variance (ANOVA) followed by Tukey’s post hoc test was used for comparisons of continuous data among multiple groups. A p value of 0.05 was considered to indicate statistical significance (*P* < 0.05).

## Results

### Cognitive dysfunction in diabetic mice

**Fig. 3:**
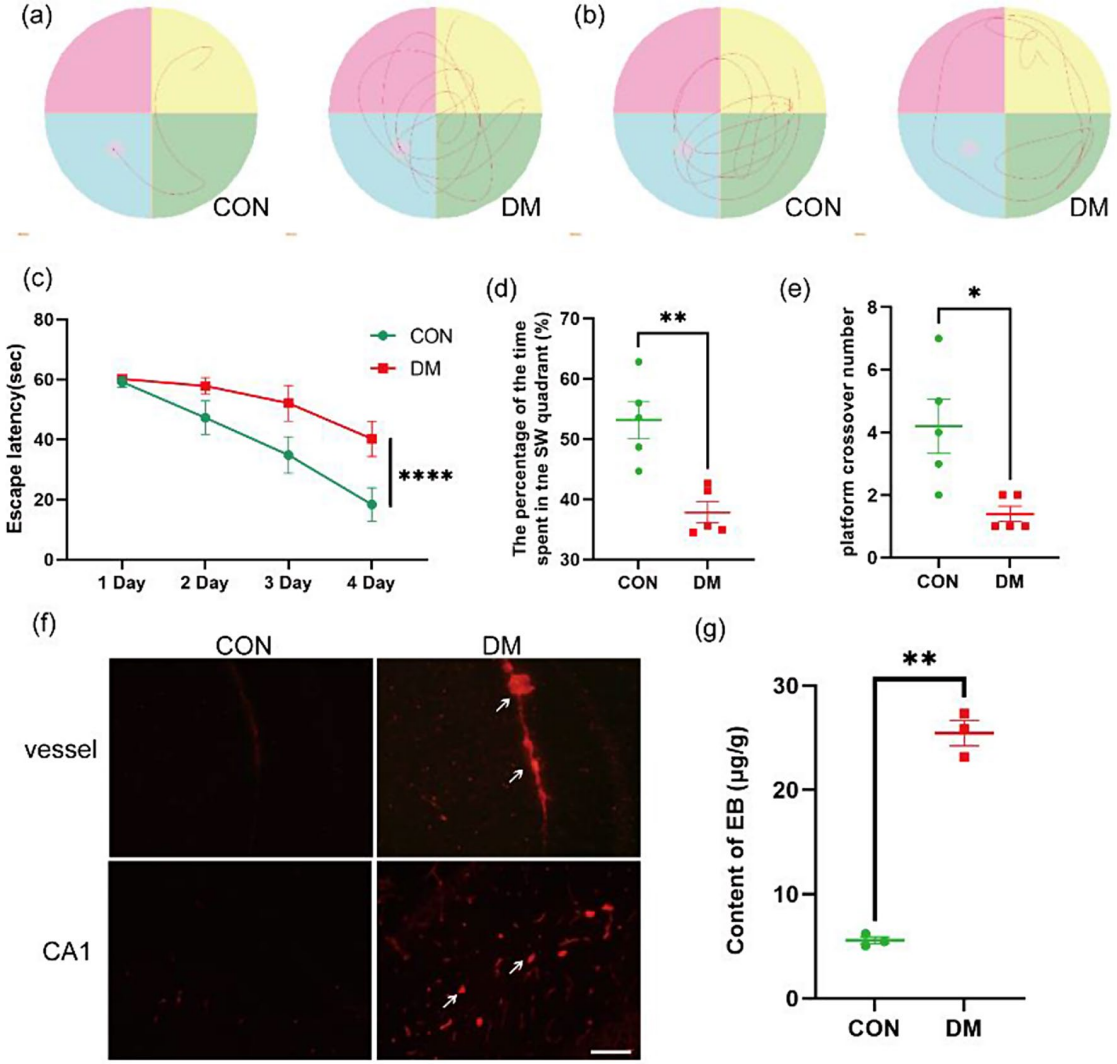
DM mice have decreased learning and memory abilities and disrupted BBB integrity. (**a**) Trajectory in the hidden platform test. (**b**) Trajectory after platform removal. (**c**) Escape latency. (**d**) Time spent in the target quadrant. (**e**) Number of platform crossings. (**f**) Representative image of EB extravasation; red fluorescence represents extravasated EB. Scale bar: 100 μm. (**g**) The content of EB was measured with a spectrophotometer. The data are expressed as the mean ± SEM (*n* = 3). * *p* < 0.05, **p < 0.01 versus the control group

At 5 w after modeling, the blood glucose levels of the mice in the DM group were elevated to a significantly greater extent than those of the mice in the control group (*P* < 0.01; Table 1), and body weight began to decrease slowly after 11 weeks of modeling. The MWM test was conducted to evaluate learning and memory. Compared with control mice, DM model mice had more difficulty finding the hidden platform (Fig. 3a). A significantly longer escape latency and fewer platform crossings were observed in the DM group than in the control group (*P* < 0.01; Fig. 3c, e). On the 5th day of the spatial exploration test, the mice in the control group were able to quickly find the platform, whereas the DM model mice swam around the pool and found it difficult to find the platform; moreover, the DM model mice spent significantly less time in the target quadrant than the control mice (*P* < 0.01; Fig. 3b, d). EB, an azo dye with a molecular weight similar to that of serum proteins, has a high affinity for plasma proteins in blood and does not readily pass through the intact BBB under physiological conditions; however, when the integrity of the BBB is impaired under pathological conditions, the amount of EB that passes through the BBB into brain tissue is increased. The exogenous tracer EB was injected into the tail vein to observe changes in the function and structural integrity of the BBB in mice. Red fluorescence in brain tissue sections was observed by fluorescence microscopy, and the EB content in the brain tissue homogenates was determined by a spectrophotometer. The results showed that the DM group exhibited significantly greater EB leakage in the hippocampus than did the control group (*P* < 0.01; Fig. 3f, g). These findings indicate that the DM model was successfully established and that deficits in learning and memory and disruption of BBB integrity were observed.

**Table 1:** Blood glucose levels and body weights of C57BL/6 mice

	Blood glucose level (mmol/L)			Body weight (g)		
	0 d	5 w	11 w	0 d	5 w	11 w
CON	5.35 ± 0.15	5.62 ± 0.28	5.84 ± 0.43	21.56 ± 0.53	25.17 ± 1.51	30.37 ± 3.05
DM	5.62 ± 0.35	21.35 ± 0.65^**^	25.64 ± 0.77^**^	22.38 ± 1.38	31.51 ± 2.45^**^	28.78 ± 2.83

Comparison of blood glucose levels and weights at different times in the control and DM groups

*Note* The data are expressed as the mean ± SEM (*n* = 10). **P < 0.01, versus the control group

### Infiltration of Th22 cells into the hippocampus leads to cognitive dysfunction in mice

**Fig. 4:**
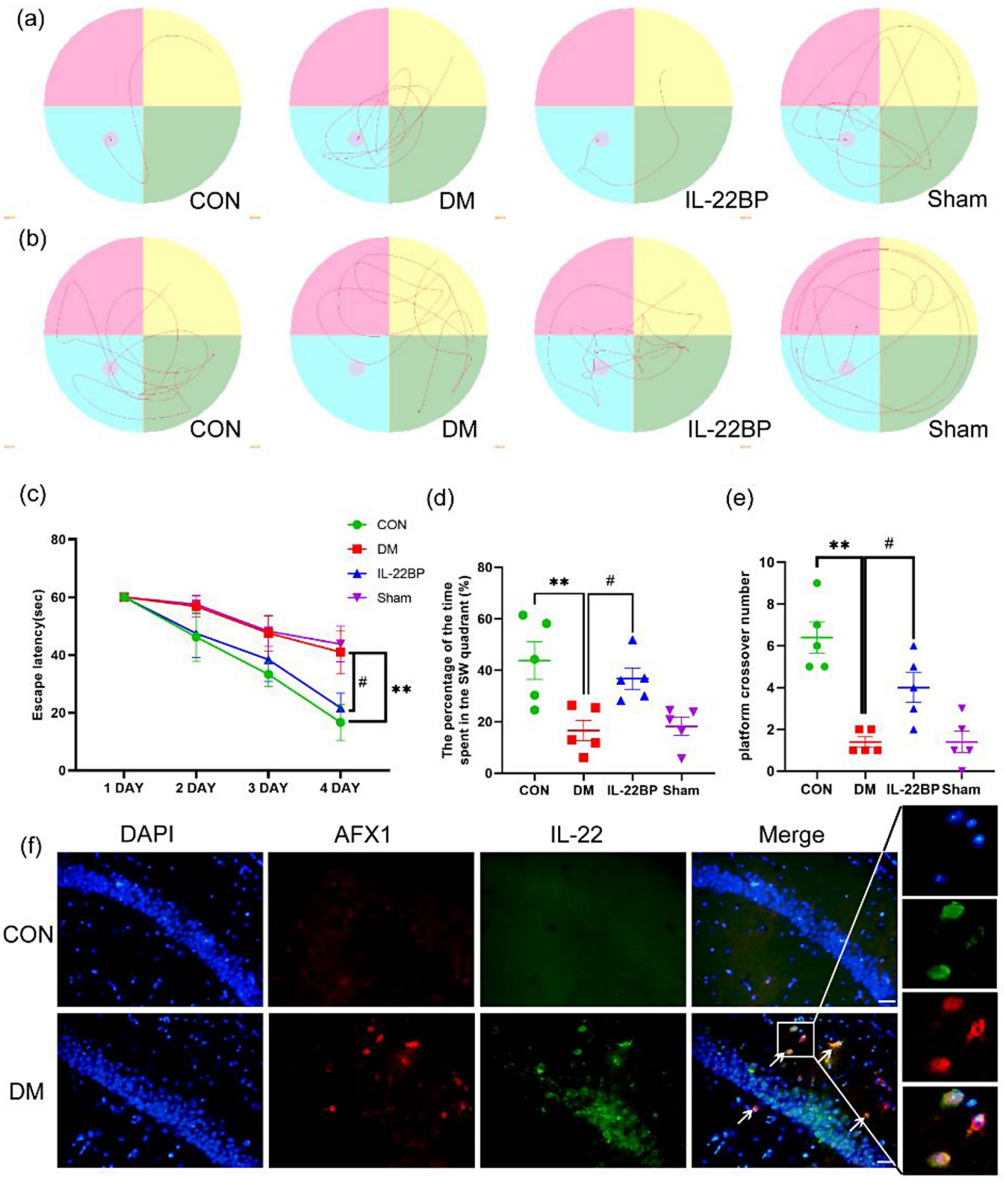
Infiltration of Th22 cells into the hippocampus leads to cognitive dysfunction in mice. (**a**) Trajectory in the hidden platform test. (**b**) Trajectory after platform removal. (**c**) Escape latency. (**d**) Time spent in the target quadrant. (**e**) Number of platform crossings. *p < 0.05 and **p < 0.01 versus the control group. ^#^*p* < 0.05 versus the DM group. (**f**) Representative image of IF staining for AFX1 and IL-22 in the control and DM groups; the arrows indicate the colocalization of AFX1 and IL-22. AFX1: red fluorescence; IL-22: green fluorescence. Scale bar: 100 μm

In the MWM test, a significantly prolonged escape latency, a decreased number of platform crossings, and a shorter time spent in the target quadrant were observed in the DM group than in the control group. Interestingly, IL-22BP ameliorated spatial learning and memory impairment induced by DM (Fig. 4a–e). To identify Th22 cells, we performed double immunofluorescence staining for IL-22 and AFX1, and the results revealed significant differences in the number of Th22 cells in the hippocampus between the DM model mice and the control mice (Fig. 4f). The above results indicate that Th22 cells pass through the BBB to enter the hippocampus in DM model mice, leading to a decrease in learning and memory abilities. However, this phenomenon can be ameliorated by IL-22BP.

### Th22 cells in the hippocampus of DM model mice induce inflammation by secreting IL-22

**Fig. 5:**
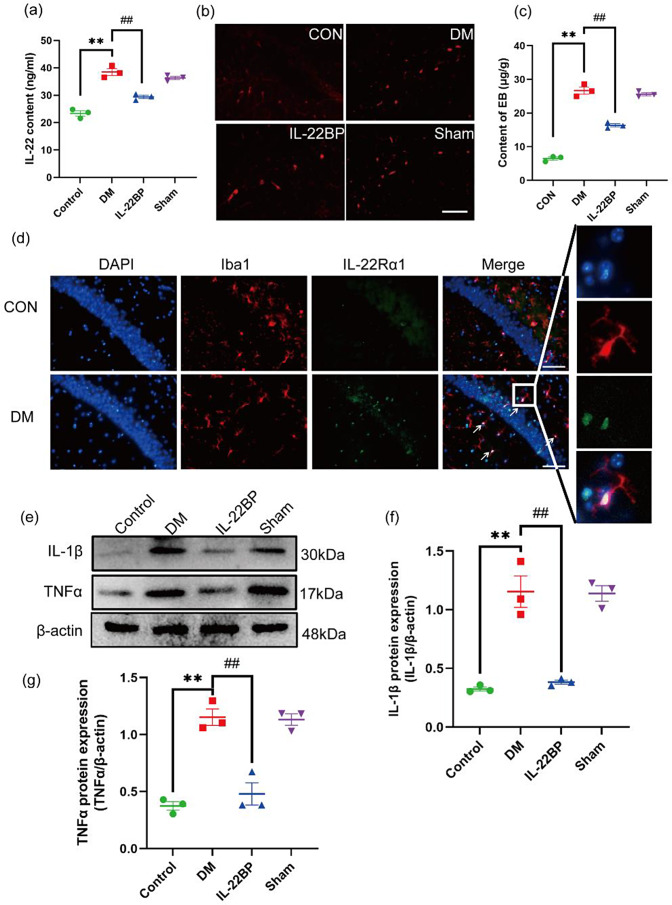
Disruption of the BBB in DM mice and increased expression of inflammatory factors induced by IL-22 secretion from Th22 cells. (**a**) The content of IL-22 was measured by ELISA. (**b**) Representative image of EB extravasation; red fluorescence represents extravasated EB. (**c**) The EB content was measured with a spectrophotometer. (**d**) Representative image of Iba-1 and IL-22Rα1 double staining in the control and DM groups; the arrows indicate colocalization of Iba-1 and IL-22Rα1. Iba-1: red fluorescence; IL-22: green fluorescence. Scale bar: 100 μm. (**e**, **f**, **g**) Western blots showing the levels of the IL-1β and TNF-α proteins in each group and quantitative analysis of the IL-1β and TNF-α protein levels. *n* = 3, *p < 0.05 and **p < 0.01 versus the control group. ^#^*p* < 0.05 and ^##^*p* < 0.01 versus the DM group

To explore the mechanism by which Th22 cell infiltration causes learning impairment, the content of IL-22 was measured by ELISA. The results showed that the content of IL-22 in hippocampal tissue from the DM group was significantly elevated. In contrast, the content of IL-22 was significantly decreased in the IL-22BP group than in the DM group (*P* < 0.01; Fig. 5a). Additionally, the EB extravasation assay showed an improvement in the integrity of the BBB after IL-22BP treatment (Fig. 5b, c). To confirm the expression of the IL-22 receptor IL-22Rα1 in the hippocampus, double immunofluorescence staining of IL-22Rα1 and Iba-1 was performed, and the results showed that IL-22Rα1 expression was increased in the hippocampus of DM model mice and that IL-22Rα1 was localized to Iba-1-expressing cells (Fig. 5d). Moreover, the WB results showed that the protein expression of IL-1β and TNF-α was increased in the DM group, while the increase in the expression of IL-1β and TNF-α was inhibited by IL-22BP administration (*P* < 0.01; Fig. 5e–g). Based on these observations, Th22 cells in DM model mice secrete the cytokine IL-22, which promotes inflammatory responses, and IL-22Rα1 is expressed on microglia.

### High glucose concentrations promote the secretion of the cytokine IL-22 by Th22 cells, leading to an inflammatory response

**Fig. 6:**
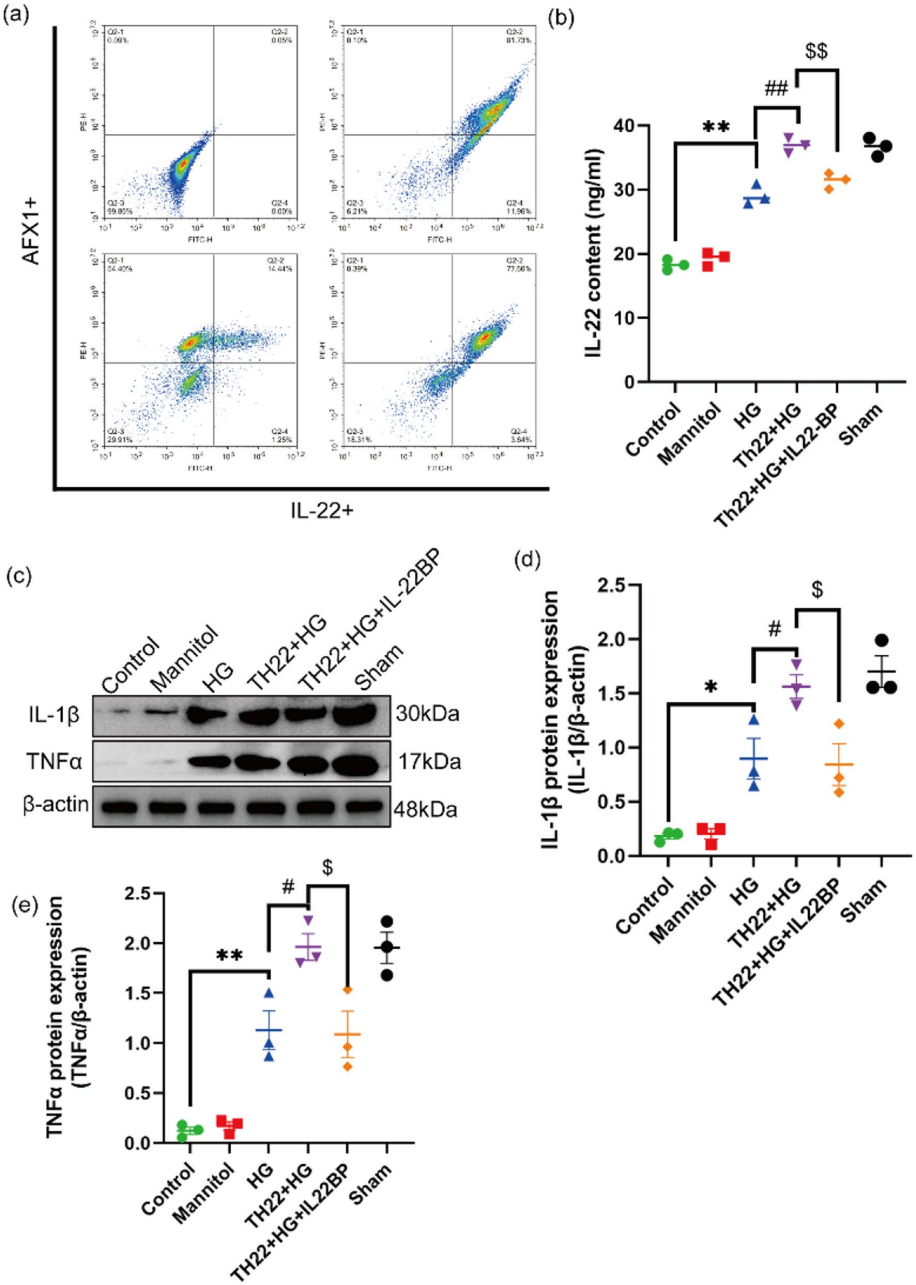
HG promotes IL-22 secretion from Th22 cells, leading to an inflammatory response. (**a**) Th22 cells were incubated with anti-IL-22-FITC and anti-AFX1-PE antibodies. (**b**) The content of IL-22 was measured by ELISA. (**c**–**e**) Western blots showing the levels of the IL-1β and TNF-α proteins in each group and quantitative analysis of the IL-1β and TNF-α protein levels. *n* = 3, *p < 0.05 and **p < 0.01 versus the control group. ^#^*p* < 0.05 and ^##^*p* < 0.01 versus the HG group. ^$^*p* < 0.05 and ^$$^*p* < 0.01 versus the Th22 coculture group

We assessed the purity of the Th22 cells by flow cytometry. Th22 cells were isolated and purified from mouse spleens and incubated with anti-IL-22-FITC and anti-AFX1-PerCP antibodies for flow cytometry. The cell purity was greater than 80%, allowing for subsequent experiments (Fig. 6a). IL-22 levels were measured by ELISA after Th22 and BV2 microglia were cocultured in vitro. The results showed that the content of IL-22 was significantly greater in the HG group than in the control group. Additionally, the content of IL-22 was significantly greater in the Th22 cell coculture group than in the HG group. However, after IL-22BP administration, the content of IL-22 was significantly decreased compared with that in the Th22 cell coculture group (*P* < 0.01; Fig. 6b). Similarly, Western blot analysis revealed that IL-1β and TNF-α protein expression was increased in the HG group. Additionally, the expression of IL-1β and TNF-α was significantly greater in the Th22 cell coculture group than in the HG group. IL-1β and TNF-α protein expression was significantly decreased in the IL-22BP group than in the Th22 cell coculture group (*P* < 0.01; Fig. 6c–e). The above results indicate that the Th22 cells in the coculture group secreted significantly more IL-22 than those in the HG group, exacerbating the inflammatory response. The decrease in IL-22 expression after the administration of IL-22BP may be related to the fact that IL-22BP binds to IL-22 at the overlapping position with IL-22R, interferes with the ability of IL-22 to bind to IL-22R, and thus inhibits the expression of IL-22 and cell signaling.

### Th22 cells promote the transformation of homeostatic microglia into reactive microglia

**Fig. 7:**
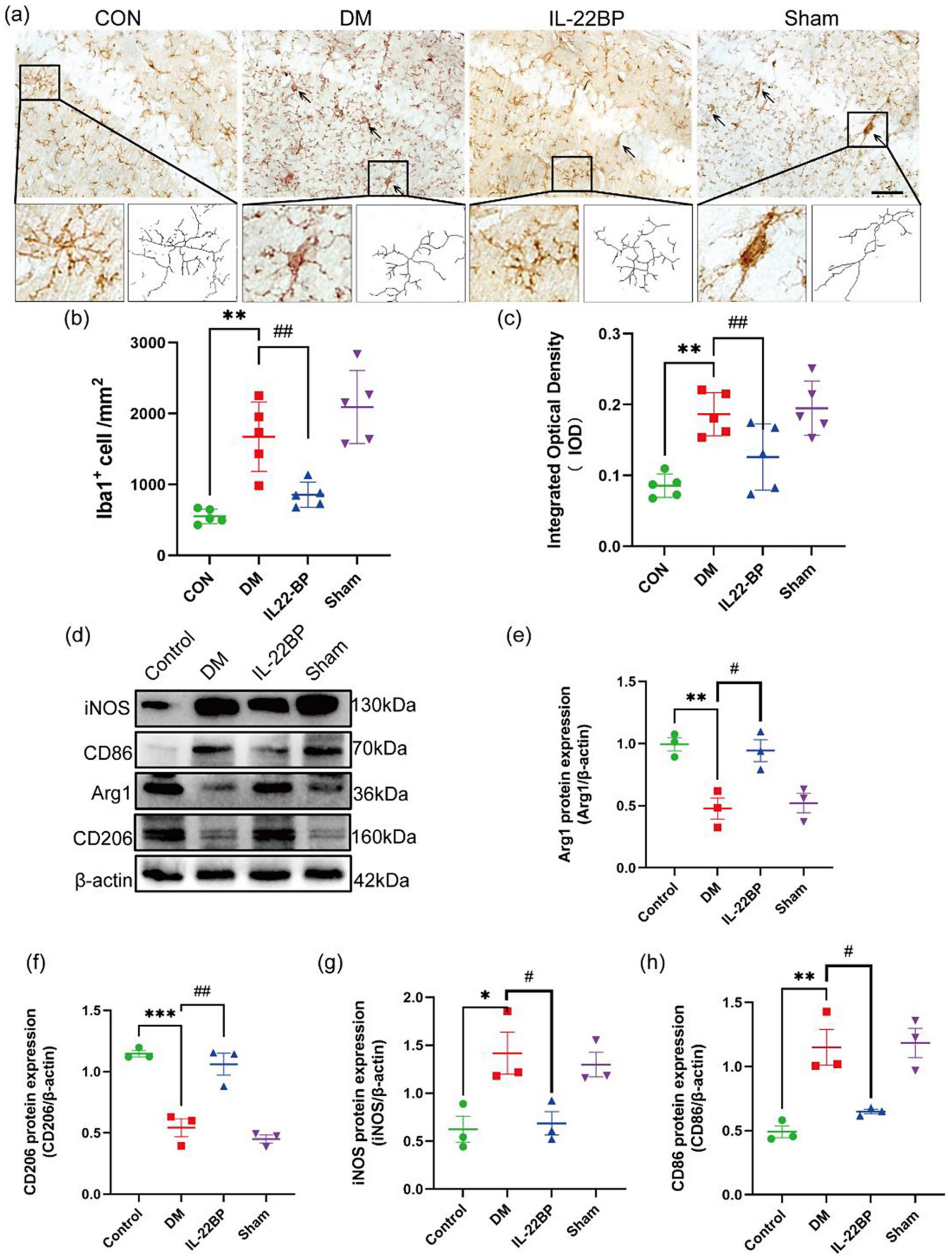
Th22 cells promote the transformation of homeostatic microglia into reactive microglia in vivo. (**a**) Representative immunohistochemical image of Iba-1 is shown. Scale bar: 100 μm. (**b**) Quantitative analyses of Iba1^+^ positive cells by number of positive cells. *n* = 5. (**c**) Quantitative analyses of Iba1^+^ positive cells in by integrated optical density. *n* = 5. (**d**–**h**) Western blots showing the levels of iNOS, CD86, Arg1, and CD206 and quantitative analysis of iNOS, CD86, Arg1, and CD206 protein expression. *n* = 3, *p < 0.05 and **p < 0.01 versus the control group, ^#^*p* < 0.05 and ^##^*p* < 0.01 versus the DM group

To investigate the effect of Th22 cells on microglial status, the morphology of microglia and the expression of related proteins were assessed both in vivo and in vitro. Immunohistochemical staining revealed that the DM group exhibited a series of changes in microglial morphology, such as shorter and thicker processes, enlarged cell bodies, and less complex branches (Fig. 7a). The number and average optical density of the Iba1 + cells in the hippocampus of the DM group mice were markedly greater than those in the hippocampus of control group mice (Fig. 7b, c). Western blot analysis revealed that microglia in the DM group had significantly upregulated iNOS and CD86 expression and downregulated Arg-1 and CD206 expression; however, these changes were significantly ameliorated after IL-22BP administration (Fig. 7d–h).

**Fig. 8:**
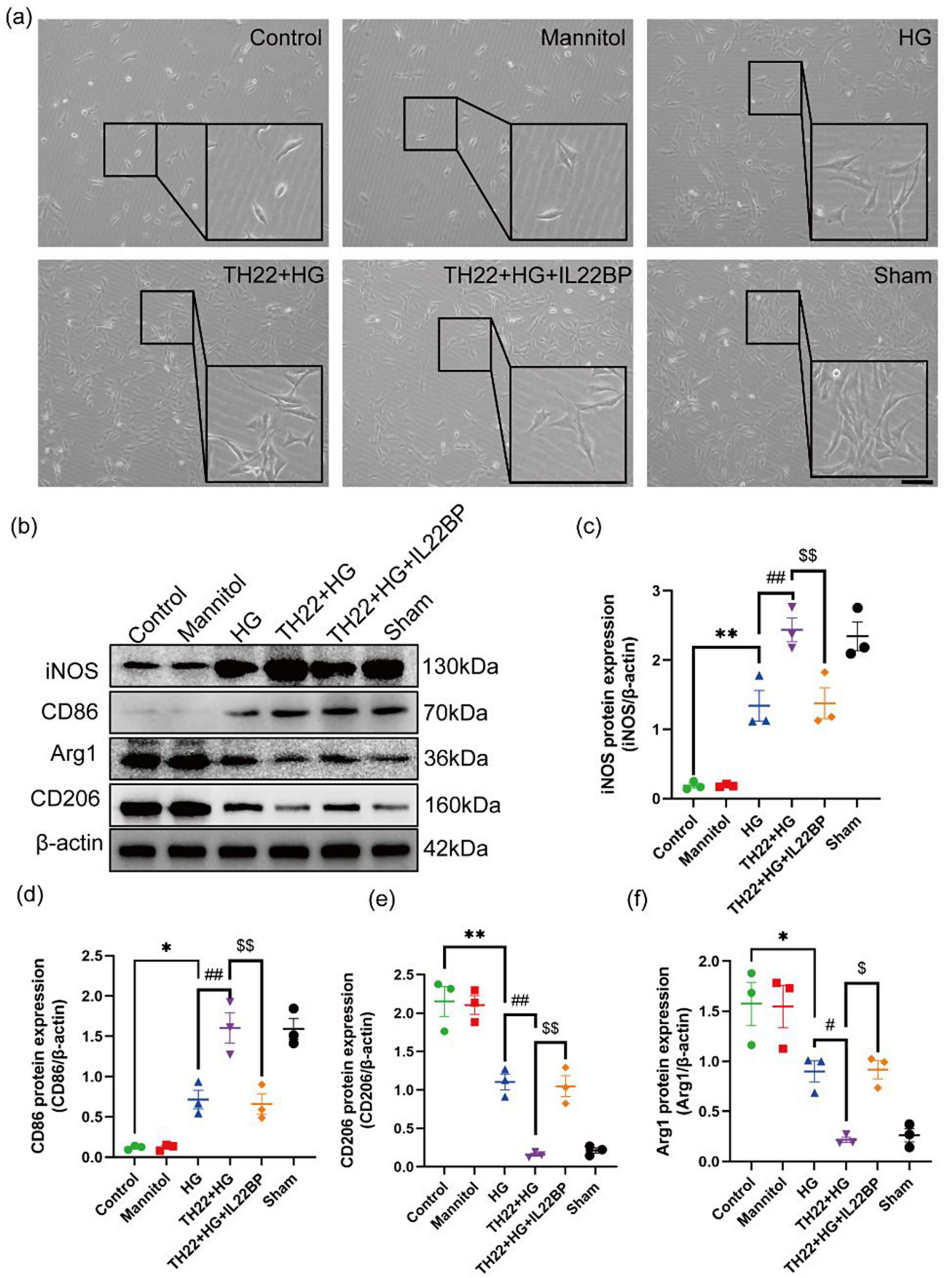
Th22 cells promote the transformation of homeostatic microglia into reactive microglia in vitro. (**a**) Representative phase contrast microscopy images of Iba-1 after 48 h of treatment. Scale bar: 100 μm. (**b**–**f**) Western blots showing the levels of iNOS, CD86, Arg1, and CD206 and quantitative analysis of iNOS, CD86, Arg1, and CD206 protein expression. *n* = 3, *p < 0.05 and **p < 0.01 versus the control group. ^#^*p* < 0.05 and ^##^*p* < 0.01 versus the HG group. ^$^*p* < 0.05 and ^$$^*p* < 0.01 versus the Th22 coculture group

**Fig. 9:**
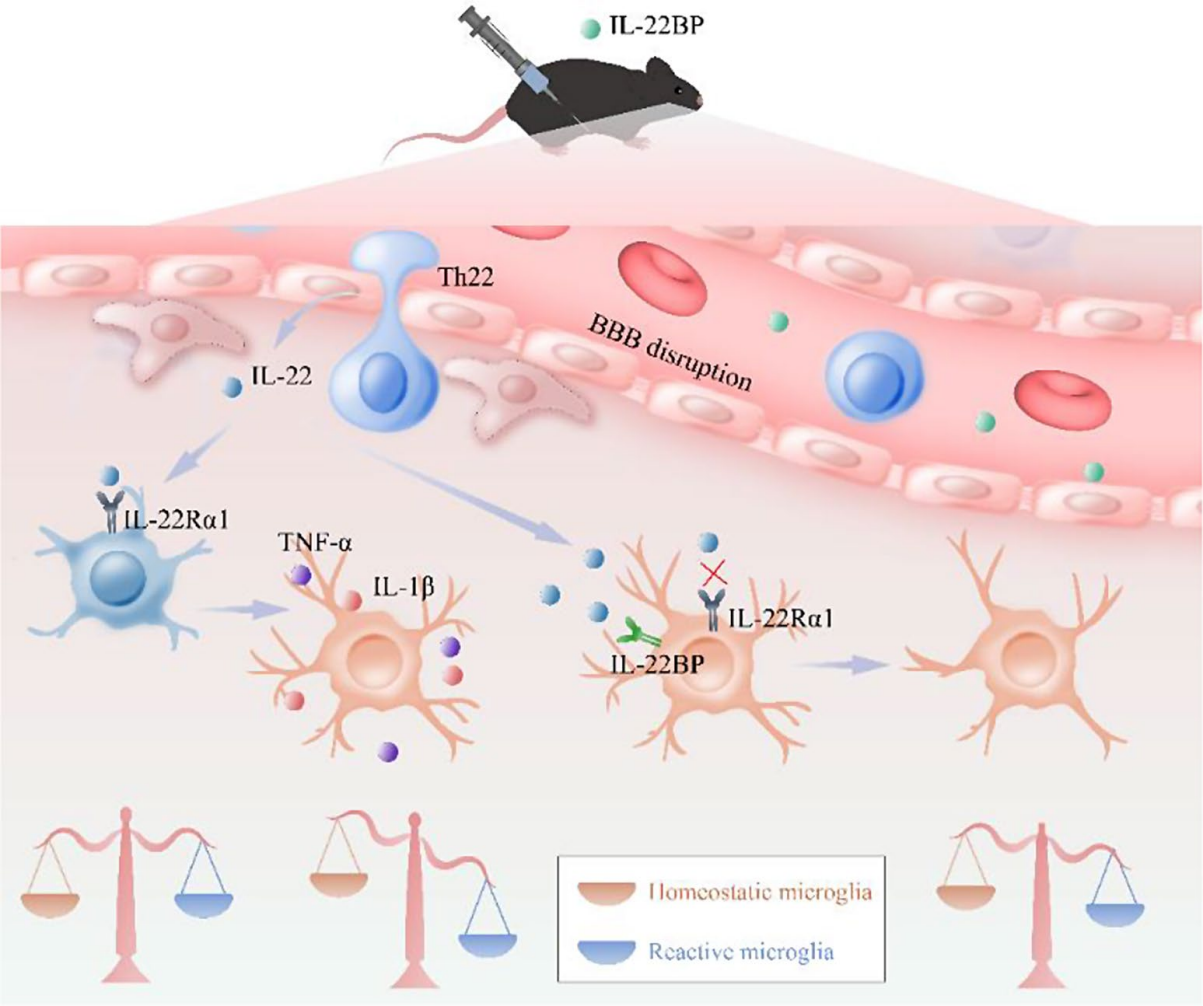
Schematic diagram showing that Th22 cells secrete IL-22 after passing through the BBB into the hippocampus, which promotes the transformation of homeostatic microglia into reactive microglia, induces an inflammatory response, and exacerbates learning and memory impairment and cognitive deficits

Similarly, after 48 h of culture, some BV2 microglia had grown in suspension, some had adhered to the wall, the cell bodies were small, the protrusions were short, and the cells mainly expressed Arg-1 and CD206 (Fig. 8a-b, e-f). After culture under HG conditions, the cell bodies were enlarged, the protrusions became longer, and the expression of iNOS and CD86 increased (Fig. 8a, b–d). The reactive microglia in the Th22 coculture group were more obvious than those in the HG group, and the expression of iNOS and CD86 was significantly increased (Fig. 8a, b–d). However, the administration of IL-22BP significantly inhibited the proinflammatory effect of the cytokine IL-22 on Th22 cells and inhibited reactive BV2 microglia (Fig. 8a–f). The above results of the in vitro experiments (Fig. 8a–f) were consistent with those of our in vivo experiments (Fig. 7a–h). In summary, IL-22 secreted by Th22 cells in the hippocampus of DM model mice alters the morphology of microglia and promotes the transformation of homeostatic microglia into reactive microglia. Interestingly, IL-22BP significantly inhibited this transformation of microglia (Fig. 9).

## Discussion

The present study indicates for the first time that the infiltration of Th22 cells into the hippocampus in a DM model results in cognitive dysfunction and the secretion of the inflammatory cytokine IL-22 to promote the inflammatory response. Th22 cells initiate the microglial response through the cytokine IL-22, resulting in a shift from microglial homeostasis to reactivity. However, the administration of IL-22BP inhibits IL-22 secretion by Th22 cells, attenuates cognitive dysfunction, reduces inflammatory responses, and inhibits reactive microglia. Collectively, these findings indicate that Th22 cells promote changes in the reactivity of microglia by secreting IL-22 in DM model mice, which participates in and accelerates the progression of cognitive dysfunction in diabetic encephalopathy.

Type 2 diabetes mellitus (T2DM) is a metabolic disorder caused by impaired insulin secretion and impaired tissue sensitivity to secreted insulin, resulting in chronic hyperglycemia [36]. T2DM is associated with metabolic syndrome, a cluster of interconnected lifestyle-related clinical features consisting of elevated fasting plasma glucose, increased blood pressure, reduced HDL cholesterol, increased circulating triglycerides, and obesity [37]. Persistent hyperglycemia, excess weight, and hyperlipidemia are related to cognitive dysfunction. In this study, we observed that DM model mice experienced increased blood glucose levels and body weight after modeling, and began to lose weight slowly at 11 weeks after modeling. These results indicated the successful establishment of a T2DM mouse model. Elevated blood glucose levels impair the integrity of the BBB, and large amounts of inflammatory factors enter the brain, leading to neurodegeneration and cognitive dysfunction [38, 39]. A close correlation between BBB damage-induced memory deficits and neuroinflammation has also been demonstrated in another type 1 diabetic stroke model [40]. In our experiments, we found that the hippocampal tissues of control mice in a normal physiological state had low EB content, indicating that BBB permeability was normal. In contrast, the hippocampal tissue of mice in the DM group showed significant extravasation of EB, and the EB content was significantly elevated. After administration of IL-22BP, EB extravasation decreased, and the EB content increased slightly. The experimental results showed that the BBB function of the DM model mice was impaired, the levels of harmful substances and macromolecular substances in the blood preventing them from entering the brain were greatly reduced, and the IL-22BP group exhibited protection against damage to BBB structure and function. The MWM test is used to assess hippocampus-related spatial learning and memory, and our results showed that DM model mice exhibited a longer escape latency, decreased number of platform crossings and shorter time spent in the target quadrant. Interestingly, after administration of IL-22BP resulted in a significant improvement in performance in the MWM test, as described above. The above results indicated that BBB integrity in DM model mice was impaired and that the mice exhibited impaired long-term spatial memory and exploration. These effects were alleviated by IL-22BP.

Necrosis following physical trauma, infection, or injury activates innate immunity [41–43]. Previous studies have shown that stress triggered by cerebral ischemia‒reperfusion injury leads to the infiltration of CD4^+^ T cells into the cerebral cortex and hippocampus [44]. Similarly, in Alzheimer’s disease, CD4^+^ T cells pass through the BBB into the brain [45]. A study on DM revealed that Th17 cells secrete IL-17 to accelerate DM development [46]. In addition, the number of peripheral blood Th22 cells is significantly increased in patients with T2DM [47, 48]. These findings reveal that CD4^+^ T cells play a key role in the pathogenesis of DM. These findings are similar to the results of our study, in which DM disrupted the BBB, leading to the infiltration of Th22 cells into the hippocampus. Th22 cells are a newly discovered group of CD4^+^ memory T cells, and studies have shown that the role of Th22 cells in chronic inflammatory diseases is closely related to the physiological function of IL-22, the major cytokine produced by these cells [49, 50]. IL-22 belongs to the IL-10 family of cytokines and exerts its effects by binding to a heterodimeric receptor complex composed of two chains, IL-22R1 and IL-10R2 [51]. Previous studies have demonstrated the involvement of IL-22 in skin tissue defense and wound healing mechanisms, as well as its protective role in intestinal infections and liver injury [52, 53]. IL-22 is expressed at elevated levels in primary Sjogren’s syndrome patients and plays a proinflammatory role in promoting salivary gland inflammation early in the disease course [54]. IL-22 plays a protective role in myasthenia gravis [55]. These findings demonstrate the dual protective and pathogenic roles of IL-22 in autoimmune diseases and inflammation. This dual role depends on the type of inflammatory response, including the type of tissue, cytokine, and inflammatory mediator and their targets of action and duration of action. It is well known that persistent inflammation caused by the secretion of large amounts of proinflammatory factors is an important cause of cognitive impairment in patients with T2DM [2].

TNF-α and IL-1β are potent proinflammatory cytokines that induce neuronal death and cause sensory and cognitive deficits by mediating peripheral synaptic instability, whereas the inhibition of TNF-α and IL-1β has been associated with the alleviation of cognitive dysfunction [56, 57]. ELISA revealed increased IL-22 and increased TNF-α and IL-1β in T2DM model mice and decreased IL-22 and inflammatory factor levels after the administration of IL-22BP. Previous studies have demonstrated that T2DM triggers the secretion of TNF-α and IL-1β by M1 microglia and the activation of the NLRP3 inflammasome, exacerbating susceptibility to CNS inflammation [58, 59]. Although the IL-22 receptor subunit IL-22 Rα1 is known to be expressed on the surface of astrocytes, endothelial cells, epithelial cells (intestinal, respiratory), and fibroblasts in some organs [60, 61], whether this protein is expressed on microglia is unclear. We hypothesized that IL-22 acts on microglia in the hippocampus to induce an inflammatory response in these cells by binding to IL-22Rα1 on their surface; this hypothesis was tested by double immunofluorescence staining for IL-22Rα1 and Iba-1. However, microglia act as innate immune cells, and the effect of Th22 cells on microglia is unclear.

Our research revealed that IL-22Rα1 is expressed on microglia in the hippocampus of T2DM mice. In addition to binding to the IL-22 receptor, IL-22 binds to the IL-22BP receptor, which is a soluble neutralizing IL-22 ‘receptor’ distinct from IL-22R1 and IL-10R2 and is encoded by a separate genus locus. Three splice variants, IL-22BPi1, IL-22BPi2, and IL-22BPi3, exist in humans. IL-22BPi2 has a greater affinity for IL-22 than for the membrane-bound receptor IL-22R, and IL-22BPi3 is able to neutralize IL-22 activity but has a lower affinity than does IL-22BPi2 but a greater affinity than does IL-22R. IL-22BPi1 cannot neutralize IL-22, is not efficiently secreted, and is partially degraded by the proteasome. 22BPi1 cannot neutralize IL-22, cannot be secreted efficiently, is partially degraded by the proteasome, is heavily retained in the endoplasmic reticulum, and induces the misfolded protein response. Only one IL-22BP isoform, similar to IL-22BPi2, exists in mice. IL-22BP acts as a glycosylated decoy secreted via the Golgi apparatus [17]. Extracellular IL-22BP targets IL-22 with high affinity, thereby inhibiting receptor activation and the biological functions of the cytokine. In vitro studies have shown that IL-22BP significantly inhibits IL-22-induced activation of the STAT3 pathway in Huh-7 and HepG2 cells, and IL-22-induced activation of STAT3 is completely blocked when the IL-22BP/IL-22 ratio reaches 10 or 50 or higher [62]. In the absence of IL-22BP, IL-22-induced uncontrolled cell proliferation may lead to tumor formation in the repair phase of colitis-associated cancer [63]. These findings are consistent with the findings of our study showing that after IL-22BP was administered in vivo, BBB integrity in T2DM model mice improved, EB extravasation decreased, EB content decreased, the expression of IL-22 and inflammatory factors decreased, spatial memory and exploratory behavior significantly improved, etc., and that the proinflammatory effect of IL-22 was inhibited by IL-22BP.

IL-22 promotes inflammation by binding to IL-22R to induce signal transduction and activators of Janus kinase 1 (JAK1) transcriptional protein 3 (STAT3) and STAT5 pathways, as well as MAP kinase pathways such as the extracellular signal-regulated kinase (ERK1/2) c-Jun Activation of N-terminal kinase (JNK) and p38 pathways. To our knowledge, microglia are local effector cells of innate immunity; once stimulated, they change their shape and function and become the main source of inflammatory cytokines, thereby increasing the inflammatory process in the brain. In diseases such as Alzheimer’s disease (AD), Parkinson’s disease (PD), glaucoma [33], and spinal cord injury (SCI), stable microglia transform into reactive microglia, resulting in the excessive production of proinflammatory factors and irreversible damage to neuronal cells [33, 57, 58]. Microglia change their morphology and phenotype to enter a responsive state during pathophysiological brain injury. Microglia in a responsive state undergo retraction, exhibit enlarged cell bodies, and proliferate at the site of brain injury [18, 22, 25]. In vivo, we confirmed that the microglia in the hippocampal tissue of the control group were round with small cell bodies and branched protrusions, while the microglia in the hippocampal tissue of the DM model mice had enlarged cell bodies, short protrusions and few complex branches and transformed from homeostatic microglia to reactive microglia. Reactive microglia release various bioactive molecules, including nitric oxide and reactive oxygen species, causing neuronal death and chronic inflammation, and these changes are associated with neuroinflammation caused by excessive production of IL-22. iNOS and CD86 are highly expressed in reactive microglia and have proinflammatory effects, while Arg-1 and CD206 are highly expressed in homeostatic microglia and have anti-inflammatory effects [64–66]. Our results showed that microglia in the hippocampus of control mice expressed high levels of CD206 and Arg-1, while microglia in the hippocampus of T2DM model mice expressed high levels of iNOS, CD86 and inflammatory factors. In addition, IL-22BP acts as a glycosylated decoy that is secreted via the Golgi apparatus. Extracellular IL-22BP targets IL-22 with high affinity, thereby suppressing receptor activation and the biological functions of the cytokine. In the present study, we found that IL-22BP significantly inhibited the transformation of homeostatic microglia to responsive microglia in DM model mice. These results are consistent with microglial changes in diabetic neuropathy [67].

To further elucidate the effects of Th22 cells on microglia in a high-glucose environment, we extracted mouse splenic CD4^+^ T cells and induced Th22 cell differentiation with IL-6 and TNF-α. There is increasing evidence that high glucose levels alter microglial activity, leading to the secretion of inflammatory factors (e.g., cytokines, cytotoxins, and ROS) [68, 69]. As a result, microglia are constantly exposed to inflammatory factors, which induces microglial reactivity via a neuroinflammatory process, ultimately triggering neuronal injury [70]. Hsieh investigated the effects of acute glucose fluctuations on BV2 microglial activity and reported that acute glucose fluctuations affect microglial activity through the MAPK, PI3K/Akt, and NF-κB cascades, leading to inflammatory activation and cellular autodegradation [71]. Our results showed an increase in the number of reactive microglia with enlarged cell bodies, prolonged protrusions and increased expression of IL-1β and TNF-α under HG conditions. After coculture with Th22 cells, the number of reactive microglia was further increased and the cells exhibited enlarged cell bodies and more elongated protrusions, which indicated that coculture with Th22 cells promoted microglial transformation into reactive microglia and the secretion of more inflammatory factors than culture alone. These in vitro experiments (Fig. 8) are consistent with previous in vivo experiments (Fig. 7) in which microglia exhibited changes. In the diabetic state, homeostatic microglia are transformed to reactive microglia with increased expression of proinflammatory factors, and IL-22BP inhibits the transition of microglia to reactive microglia and suppresses the expression of inflammatory factors. These results suggest that the inhibition of IL-22 contributes to the attenuation of microglia-mediated inflammatory responses.

In addition, we found that IL-22 secretion by Th22 cells through the BBB induced alterations in microglial cell morphology and function, whereas the administration of IL-22BP to mice inhibited reactive microglia-mediated attenuation of cognitive dysfunction caused by DE, suggesting that IL-22BP may be a potential therapeutic target for DE. Neutralizing Abs against IL-22 are being investigated for the treatment of psoriasis [72]. In a mouse model of sepsis, injection of IL-22BP-Fc reduced disease severity [73]. Conversely, pharmacologic modification of IL-22BP may be an effective strategy for alleviating cirrhosis [74]. Despite the importance of endogenous IL-22BP in the regulatory circuits controlling IL-22 activity, especially at biological barriers, excess IL-22BP above physiologic levels may have adverse effects. Decreases in bioactive IL-22 levels may lead to problems such as increased susceptibility to gastrointestinal or pulmonary infections [17]. Long-term increases in the levels of biologically active IL-22 and reductions in the levels of IL-22BP may have long-term effects on the development of colorectal carcinogenesis [75]. These issues still need to be studied in depth. There are several limitations of our study. First, this study focused on the effect of IL-22 secreted by Th22 cells that passed through the BBB and entered the hippocampus on microglia in a T2DM model, but the effect of Th22 cells on microglial cells through this signaling pathway has not been studied in depth. The next step is to investigate the signaling pathways involved in the pathogenesis of DE and the effects of Th22 cells on other cells in the brain.

## Conclusion

In conclusion, our study demonstrated for the first time that in a mouse model of DE, Th22 cells secrete IL-22 after passing through the BBB into the hippocampus and promote the transformation of homeostatic microglia into reactive microglia, which induces an inflammatory response, exacerbates learning and memory impairment and cognitive deficits, and contributes to and accelerates the development of DE. Based on our experimental results, we hypothesize that IL-22BP inhibits reactive microglia and attenuates cognitive deficits caused by DE. Although this investigation provides insightful evidence, additional comprehensive studies are needed to validate our conclusions.
